# Psychosocial Assessment Practices for Hematopoietic Stem Cell Transplantation: A National Survey Study

**DOI:** 10.21203/rs.3.rs-3044597/v1

**Published:** 2023-06-29

**Authors:** Lori Wiener, Timothy Sannes, Jill Randall, Sheila Lahijani, Allison Applebaum, Tamryn Gray, Natalie McAndrew, Benjamin Brewer, Hermioni Amonoo

**Affiliations:** National Cancer Institute; University of Massachusetts Chan Medical School; University of Michigan; Stanford University School of Medicine; Memorial Sloan Kettering Cancer Center; Dana-Farber Cancer Institute; University of Wisconsin-Milwaukee; University of Colorado Anschutz; Dana-Farber Cancer Institute

## Abstract

Psychosocial health predicts and contributes to medical outcomes for patients undergoing hematopoietic stem cell transplantation (HSCT). Yet, there are no standards for psychosocial assessments or support for both patients and caregivers across the care continuum. To examine the current state of psychosocial care, clinicians were sent a survey of their psychosocial assessment practices for patients and caregivers undergoing HSCT via the Listservs of professional organizations. Descriptive statistics and bivariate analyses were performed to summarize the findings. While 96% of participants reported routine pre-HSCT psychosocial assessment of patients, only 10.6% routinely used a validated transplant risk-assessment measure. Just 27% routinely performed follow-up psychosocial assessments. In contrast, only 47% of participants routinely assessed the psychosocial needs of family caregivers pre-HSCT, and 13% routinely performed follow-up assessments for caregivers. Most (90%) reported social workers were the primary providers of assessments. While patient-report measures were used for evaluation, the majority of assessments were clinical interviews. No significant differences were found between programs that treated adult and pediatric patients versus those only treating adult patients. Our findings highlight the lack of standard psychosocial practices for patients and family caregivers undergoing HSCT and we offer recommendations to fill this gap.

## Background

Hematopoietic stem cell transplantation (HSCT) offers a potential cure for many children and adults with hematologic disorders, metabolic disorders, and bone marrow failure syndromes.^1^ However, HSCT is intensive and entails high-dose chemotherapy with treatment-related toxicities, prolonged hospitalization and recovery, and may result in potentially life-threatening complications.^2^ Hence, patients undergoing HSCT may endure significant psychological distress throughout the transplant trajectory.^3,4^ Psychological distress in the HSCT population is multifaceted and characterized by numerous symptoms, including depression, anxiety, insomnia, fatigue, delirium, and post-traumatic stress symptoms, each associated with worse health-related outcomes, including diminished quality of life (QOL),^3,4^ functioning, and increased mortality.^5–8^

Despite well-known associations between psychological distress and outcomes in this population, there is limited data on best practices for assessing psychosocial health in the HSCT population. Distress screening is mandated for cancer program accreditation, and the National Comprehensive Cancer Network (NCCN) has provided several resources and validated standardized measures to help cancer programs effectively carry out this mandate.^9^ These screening tools are now used by most pediatric and adult HSCT centers^10^ to accurately diagnose distress in patients. Since HSCT and its recovery is intense and prolonged, systematic, prospective, and longitudinal assessments are essential to effectively characterize patients’ psychological needs for timely treatment or triage available psychosocial resources. Yet, while distress screening initiatives have increased awareness of the psychological impact of cancer more broadly, limited research exists on best practices for assessing psychosocial health throughout the transplant trajectory in both adult and pediatric populations.

Previous reports focused exclusively on pre-transplant assessment: one identified whether adult and pediatric programs require psychiatric evaluation,^11^ and another described psychosocial assessment practices in adult programs only.^12^ Some studies have found that pre-transplant psychosocial risk assessment tools such as the Transplant Evaluation Rating Scale (TERS), the Stanford Integrated Psychosocial Assessment for Transplantation (SIPAT), and the Psychosocial Assessment of Candidates for Transplant (PACT) predict post-transplant outcomes, including survival, medical adherence, delirium, ICU transfer, readmissions, hospital length of stay, and quality of life.^13–21^ Since many psychosocial risk factors (e.g., adherence to medical regimens, cognition, quality of family support) are challenging to obtain from patients’ self-report due to recall bias, these risk assessments are often completed by specialty mental health clinicians. However, there are no standards for pre-transplant assessment of psychosocial and behavioral factors or using these tools.^22^ With increasing clinical indications for HSCT for both benign and malignant diseases across the lifespan, it is important to adequately assess and manage psychological health before, during, and after transplant to promote psychological well-being and its impact on health-related outcomes. This assessment is also critical for identifying the needs of the caregivers supporting HSCT patients.

Family and friend caregivers (sometimes called “informal caregivers”) are crucial to every aspect of the HSCT trajectory.^23^ HSCT caregivers must navigate a myriad of responsibilities to support patients, including complex medication management, care coordination,^24^ and transportation of patients to and from weekly follow-up visits once the patient is discharged.^25^ Unfortunately, caregivers’ health can be compromised as they grapple with multiple responsibilities, which often add to their distress. Further, parents of pediatric HSCT recipients (i.e., parental caregivers) must learn to manage the needs of their sick child and their other children or family members, which can worsen overall distress. Not surprisingly, patient and caregiver well-being are inextricably linked.^26,27^ Adequate assessment and management of caregiver psychological health impacts both patient and caregiver outcomes.^26,27^ However, there are no established guidelines for how to approach caregiver psychosocial assessment and care throughout the HSCT trajectory.^25,28–30^

Accordingly, this study used a cross-sectional survey of HSCT programs in the United States (U.S.) to determine current practices for psychosocial assessments in patients and their caregivers prior to, during, and post-HSCT. With data from this survey, we aim to inform national guidelines and standardize psychosocial assessment for HSCT patients and caregivers.

## Methods

### Study Design

We used a web-based self-administered cross-sectional survey. The study was deemed exempt by the National Institute of Health (NIH) Office of Operation. The survey was fully anonymized, built, and collected using an external, encrypted website (SurveyMonkey). The survey began with background information and objectives related to the study. Two questions followed this introduction, “I agree to participate in this study” and “Do you treat or care for patients undergoing HSCT.” If “no” was provided for either question, the survey ended, and the participants received a “thank you” message for their time. Survey completion by participants indicated informed consent.

In September 2022 and October 2022, we sent the survey to members of the *Association of Pediatric Oncology Social Workers, the Society of Pediatric Psychology Hematology/Oncology/Bone Marrow Transplant Special Interest Group (SIG), BMTInfoNet*, and the *Exchange* (an online network of social workers hosted by the National Marrow Donor Program) via their Listservs. In January 2023, we sent the survey to members of the *Association of Oncology Social Work* through their Listserv. Each group received up to three reminders. Eligible participants were those currently treating or caring for patients undergoing HSCT. The instructions asked for one participant per institution.

### Survey Development and Methods

The survey was designed by an interdisciplinary national group of psychosocial clinicians, researchers, and leaders from diverse training and backgrounds (e.g., psychiatrists, psychologists, social workers, and nurses) who work in HSCT and are members of the Transplantation and Cellular Therapy (TCT) Special Interest Group of the American Psychosocial Oncology Society (APOS).

We designed the 28-item survey to gain a nuanced understanding of psychosocial assessments and practices in the US. Questions covered HSCT program characteristics, including location, the age range of patients transplanted, whether pre-transplant assessments are conducted, type of psychosocial provider (available for support and who conducted pre-HSCT assessments), psychosocial assessment tools administered, and overall practices for psychosocial assessments. Additionally, we included questions about the timing of assessments collected during follow-up care for patients. The same questions were asked pertaining to caregivers. The survey was beta tested by six members of the TCT SIG to assess question clarity, ease of answering questions, sequence of the questions, and to identify any omissions.

### Statistical Analysis

The majority of analyses were descriptive: summarizing the frequency of questions completed, how many responses were endorsed, and the mean and standard deviations of all continuous response items. Chi-square tests were conducted to compare responses between programs that endorsed treating patients below the age of 18 (in addition to adults) versus programs only treating patients above the age of 18 across dichotomous yes/no responses. The number of responders was used as the denominator for missing responses.

## Results

**Figure 1:**
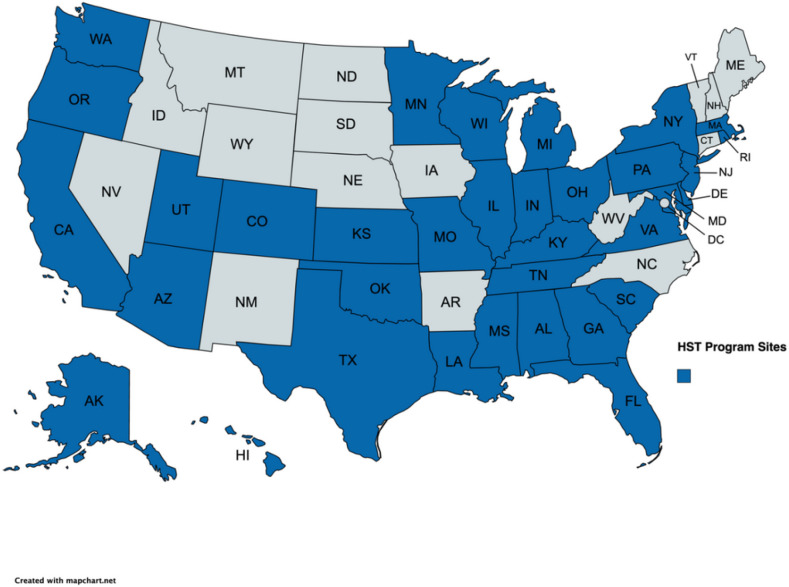
Map of the United States showing where HSCT Centers of respondents are located in the United States by State

Of the 143 respondents who opened the study link and agreed to participate, each of the survey items were completed by 79–100% of respondents (*M* = 84.5%). Participants were from 34 US states (See Fig. 1). Most respondents worked at University Hospitals (65.3%) that treated both adult and pediatric patients (56.1%). The average minimum age of patients treated was 7.82 (*SD* = 8.89), and the average maximum age of patients treated was 54.15 (*SD* = 27.16). Twelve percent of respondents did not provide transplant recipient age ranges.

### Pre-HSCT Psychosocial Screening

**Figure 2:**
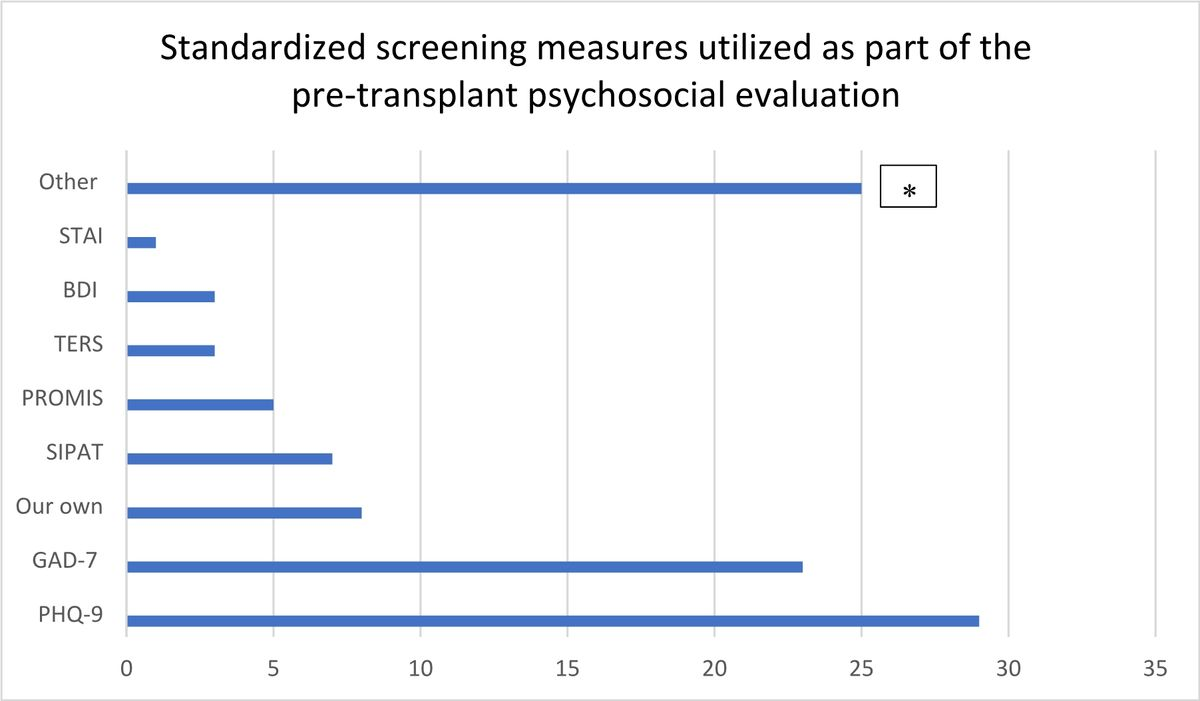
This figure shows distribution of standardized screening measures used as part of the pre-transplant psychosocial evaluation

Most respondents (96%) reported pre-transplant psychosocial evaluations were primarily completed by social workers (90%) and/or psychologists (32.2%), with assessments focusing on high psychosocial risk factors (97.5%), support systems (96.7%), patients’ concrete needs (94.2%), and understanding of the transplant process (81%). An additional 10.7% checked “other,” and the majority of the “other” responses were to assess concrete needs and coping (Table 1). Forty-four percent reported that pre-transplant assessment was conducted as a baseline measure of psychosocial needs. Pre-HSCT assessments consisted of a clinical interview (51.7%) or a combination of a clinical interview along with standardized measure(s) (45%). Standardized measures were quite varied (Fig. 2), with the Patient Health Questionnaire 9 (PHQ-9) most often utilized (59.2%), followed by Generalized Anxiety Inventory-7 (GAD-7; 46.9%). Only 10.6 percent of respondents reported using a validated risk-prediction tool in their setting, and the SIPAT and the TERS were the most commonly used. Additional standardized measures, written as “other,” are listed in Table 2. There were no differences between programs that treated adult and pediatric patients versus those only treating adults in whether they conducted pre-HSCT psychosocial screening *X*^*2*^ (1, 124) = 1.18, *p* = 0.28.

### HSCT Follow-up Psychosocial Screening

Approximately a quarter of the respondents (26.7%) reported collecting repeat/follow-up clinical assessments following pre-HSCT assessments. These follow-up assessments are administered around day 100 (23.5%), at 6 months (11.8%), and at 1 year (26.5%) post-HSCT. An additional 52.9% of respondents checked “other” for when these assessments were repeated, with most conducted at discharge and subsequent admissions. If a patient reports psychological changes, social workers continue to provide the majority of the follow-up clinical assessments (88.3%), along with psychologists (25%). There were no differences between programs that treated adult and pediatric patients versus those only treating adults in whether they conducted follow-up psychosocial screening *X*^*2*^ (1, 114) = 6.18, *p* = 0.43.

### Caregiver Psychosocial Screening

Approximately half of the respondents (49.1%) reported conducting caregiver assessments, and these were primarily completed by social workers (88.3%) and/or psychologists (25%). Psychosocial caregiver screening was almost exclusively done via clinical interview (96.3%), with only 5.4% including any standardized measures. The majority of respondents (94.6%) documented the results of the caregiver screening in the patient’s electronic medical record. There were no differences between programs that treated adult and pediatric patients versus those only treating adults in whether they conducted caregiver psychosocial screening *X*^*2*^ (1, 115) = 0.76, *p* = 0.38.

### Caregiver Follow-up Psychosocial Screening

Less than a quarter (N = 13, 23.3%) of respondents reported conducting follow-up caregiver assessments during or post-transplant. Caregiver assessments occurred at 100 days following the transplant (N = 4, 28.6%) and one year (N = 4, 28.6%). There were no differences between HSCT programs that treated adult and pediatric patients versus those only treating adults in whether they conducted follow-up caregiver screening *X*^2^ (1, 56) = 2.27, *p* = 0.13.

### Psychosocial Support Services Provided During Transplant

Routine follow-up support was mostly provided as “the needs arise” (69.8%) though some reported having a set time point for further assessment (26.7%). If the transplant recipient requested psychosocial support services, these were provided by both social work (48.3%), psychology (25%), psychiatry (2.6%), or “other” (24.1%). “Other” services reported in open text included palliative care, child life specialist, art and/or music therapist, or chaplain. Support consisted of individual counseling (93.9%), peer support (45.2%), support groups (44.4%), family counseling (43.5%), and “other” (20.9%). “Other” reported support services included referrals to community programs, psychiatry, sibling programs, and art/music/massage therapies. Following day 100, 69.6% of respondents reported that transplant recipients received support only “if the need arises.” Less than a quarter of the transplant patients (22.3%) were routinely scheduled to meet with palliative care, as opposed to if clinically indicated. Those who had met with palliative care clinicians did so prior to the transplant or when first admitted for conditioning. Programs that treated adult and pediatric patients were more likely to routinely offer palliative care services than those only treating adult patients *X*^2^ (1, 113) = 7.27, *p* = .007.

Less than half of caregivers (43.9%) were offered psychosocial support during the transplant trajectory. HSCT programs provided even less systematic support for caregivers after day 100 post-transplant (21.9%). When caregivers received support, it was predominately individual counseling (65.7%), support groups (38.9%), family counseling (30.6%), and couples counseling (14.9%) provided by social workers.

## Discussion

This interdisciplinary national survey study explored psychosocial assessments and support provided to patients and their caregivers before, during, and after HSCT at pediatric and adult centers. We found that almost all sites include a psychosocial assessment pre-transplant, most often administered by a social worker. Areas prioritized for assessment include: identifying high psychosocial risk factors, current support systems, patients’ concrete needs, and their understanding of the HSCT process, with a limited offering of palliative care services to patients. Half of the pre-HSCT assessments consisted of a clinical interview only, and half consisted of a combination of a clinical interview and standardized measure(s). While close to half of the respondents reported a plan to repeat baseline psychosocial assessments throughout HSCT, only a quarter completed repeat/follow-up clinical assessments. In contrast to previous findings that psychosocial professionals rated assessing the caregiver as one of the most important aspects of the pre-HSCT psychosocial assessment,^31^ our study found that only 49% of programs administer caregiver psychosocial assessments pre-transplant. Furthermore, only a quarter administer follow-up assessments for caregivers.

We show that pre-HSCT psychosocial assessments were more routine compared to follow-up or longitudinal assessments. Several studies highlight that psychological distress can begin at any point in the HSCT trajectory and worsen over time.^32,33,34^ Hence, a one-time cross-sectional assessment will not capture the changing psychosocial needs of patients and caregivers throughout HSCT recovery, contributing to unmet psychosocial needs. Although we did not obtain explicit information about the lack of follow-up care, persistent shortages of specialty mental health clinicians^35^ and inadequate clinical social work staffing levels^36^ are likely contributing factors. Additional factors not captured from our survey, such as insurance requirements that drive the predominance of pre-HSCT assessments, may also provide clues.^37–39^ With the increased use of electronic patient-reported outcome (e-PROs) measures in diverse oncological populations,^40–42^ more work is needed to characterize how e-PROs could facilitate a more longitudinal assessment of psychosocial needs in the pediatric and adult HSCT population.^39,43^

Standardized psychosocial assessments for HSCT recipients and their caregivers are lacking.^12^ Clinicians who completed our survey reported using 23 different measures, with the Patient Health Questionnaire 9 (PHQ-9) most commonly used. Despite existing validated pre-transplant psychosocial risk-prediction measures, such as the TERS, SIPAT, and PACT, very few transplant centers reported consistent use of these measures, which are powerful predictors of transplant outcomes, often showing better predictive validity than medical comorbidities for survival and other key outcomes post-transplant.^22,44–46^ Therefore, we recommend that all transplant centers incorporate the TERS, SIPAT, or PACT in all pre-transplant assessments by psychosocial assessment clinicians.

**Figure 3:**
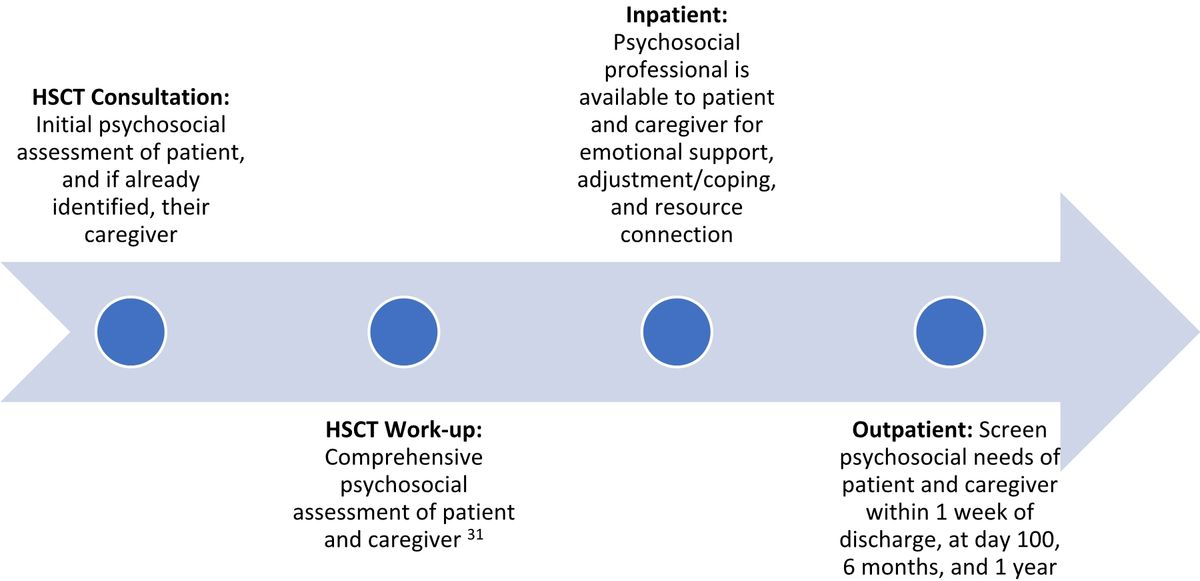
This figure provides recommendations of psychosocial assessments for consideration over the course of the transplant course

While caregivers’ availability and commitment to overseeing patient care is critical to a successful transplant,^23^ transplant centers do not routinely assess and manage caregiver psychological distress. Following the pre-transplant workup, less than a quarter of caregivers were re-assessed at any time. Most of these assessments were not completed until 100 days post-transplant, typically at the end of the formal caregiving period. When provided, most interventions given were individual counseling. One way to improve caregiver assessment and intervention may be through improved documentation. We found that documentation of caregiver assessments and interventions was predominantly under the patient’s medical record number (MRN). There is a push for caregivers to have their own medical record number (MRN).^47^ Caregiver medical record numbers identify caregivers in greatest need of support, help coordinate their care,^47,48^ and establish a pathway to bill for caregivers’ own psychosocial or medical care.^47^ We provided strategies (Table 3) and a suggested timeline (Fig. 3) for implementing screening and support for patients and caregivers throughout the transplant trajectory.

Participants reported that palliative care is infrequently offered to HSCT recipients despite robust evidence for the benefits of palliative care on various clinical outcomes in oncological populations, including depression and anxiety symptoms, physical symptom burden, and mortality.^49–52^ Although randomized trials of integrated palliative care for the HSCT population show improved outcomes,^53–55^ palliative care use remains limited. Our results indicated that programs treating adult and pediatric patients were more likely to incorporate palliative care services than those only treating adult patients. This may be in concert with national surveys in which pediatric transplant programs overwhelmingly support the integration of palliative care.^56^ Persistent shortages of palliative care clinicians and cultural perceptions of palliative care as synonymous with end-of-life care^57^ may contribute to inadequate utilization of palliative care for HSCT recipients. We recommend integrating psychosocial and palliative care resources embedded in the HSCT team, where they can work together while maintaining longitudinal relationships with patients, caregivers, and medical providers.

There are several significant limitations that warrant discussion. First, we could not determine a traditional response rate due to our use of Listservs to recruit participants and the possibility that some respondents may have been listed in multiple Listservs. Second, although we asked participants to provide one respondent per site, this was not guaranteed. Third, we were unable to determine whether transplant centers exclusively treated pediatric or adult patients due to the wording regarding patients’ age ranges, which limited our ability to conduct subgroup analyses. Fourth, all question responses were optional, with skip patterns built in, and not all participants answered every question, resulting in missing data and possible bias in interpreting the findings. Future surveys may benefit from more in-depth inquiry about patient socioeconomic status, diversity, languages spoken, and the availability of validated tools for diverse languages. Cost analyses may be beneficial in depicting the relatively low cost and sustainability of validated psychosocial assessments that mitigate response burden while also reducing or preventing mental health care costs following HSCT for patients and their caregivers. Additionally, the fiscal benefits of psychosocial assessments in HSCT populations may result in increased palliative care service, social work, and mental health care provisions.

In conclusion, this study confirms the lack of standard practice for psychosocial assessment and support for patients undergoing HSCT and their caregivers in the U.S. We recommend the routine use of validated risk-prediction tools for all pre-transplant risk assessments, that caregivers and patients be assessed or screened for distress and other needs prior to transplant and at critical time points post-transplant up to and including at one year post. We suggest that psychosocial and palliative support be embedded into the transplant team to enhance clinical support during the transplant trajectory. A comprehensive understanding of the psychological and mental health needs of patients undergoing HSCT and their caregivers allows for more tailored care and improved outcomes.

## Figures and Tables

**Table 1:** “Other” items included in pre-HSCT assessments

Items	Number of times reported
Coping (ability to adhere to medications, self-efficacy, high-risk behaviors, medical trauma history)	6
Additional support needs (lodging, financial needs, prescriptions)	6
Insurance (pre-certification, approval, for post-HSCT)	4
Advance Directives	2
Sexuality, sexual health	1

**Table 2:** Additional Measures Used in HSCT Assessments

	Participants that reported using the measure (N)
*Adult Measures*	
Montreal Cognitive Assessment (MoCA)^58^	6
Alcohol Use Disorders Identification Test (AUDIT)^59^	5
Psychosocial Assessment of Candidates for Transplant (PACT)^21^	3
Brief COPE^60^	2
Functional Assessment of Cancer Therapy (FACT)-BMT	2
NCCN Distress screening	2
FACIT Measure of Financial Toxicity (FACIT-COST)^61^	2
Brief Medical Numbers Test (BMNT)^62^	1
Multidimensional Health Locus of Control (MHLC)^63^	1
Beck Anxiety Inventory^64^	1
Mini Mental State Exam (MMSE)^65^	1
World Health Organization measuring Quality of Life (WHOQOL-BRIEF)^66^	1
*Pediatric Measures*	
Peds QL^67^	2
Psychosocial Assessment Tool (PAT)^68^	2
PTSD Screener	1
Transition Readiness Assessment Questionnaire (TRAQ) (AYA)^69^	1
Symptom Assessment Pediatrics Tool (SSPedi)^70^	1
Wechsler Intelligence Scale (WISC or WAIS)^71^	1
Behavior Assessment System for Children (BASC)^72^	1

**Table 3:** Recommended strategies

Method	Time Point	Recommendations
Assessment	Patient PreTransplant:	- Identify psychosocial risk factors for successful transplant outcome, including current mental health presentation/coping, psychosocial history, substance use, medical adherence, understanding/expectations of HSCT, and social support^31^ - Use a validated pre-transplant risk assessment tool (e.g., TERS, SIPAT, PACT)
	Patient Longitudinal:	- Hospital: Screened/triaged during inpatient rounds - Outpatient: Day 80–100, 6 months, 1 year post^73^ - Allows assessment of changing psychosocial needs throughout the HSCT trajectory and recovery
	Caregiver PreTransplant:	- Identify caregiver(s) availability, ability to meet anticipated needs, understanding/expectation of HSCT, psychosocial/health risk factors, ability to provide care throughout HSCT, social support - Use a validated pre-transplant risk assessment tool and or brief clinical assessment tool
	Caregiver Longitudinal:	- Within one week of patient discharge, Days 80–100, 6 months, 1 year - Allows assessment of caregiver needs throughout the HSCT trajectory - Use a validated screening tool or brief clinical assessment (e.g., PAT-HCT or CSS-CG) - Document assessments under the caregiver’s MRN
Follow-up Care	Patient support prior to HSCT: As needed, provided by psychosocial and palliative care providers	- Treat psychosocial concerns identified in the assessment that impact transplant or are related to the medical situation, provide an opportunity for advance care planning, help build a social support network to be used during and following HSCT - Psychosocial and palliative providers who are embedded in the HSCT team and integrated for appropriate triage are recommended - Consider peer-to-peer support and group modalities of intervention
	- Patient support provided during HSCT	- Tailor interventions based on an assessment of needs: individual counseling, group education/support, facilitating communication with medical team and family, palliative interventions Consider peer-to-peer support
	Caregiver Support provided during HSCT	- Interventions based on assessments - Consider individual, family, group counseling, and peer-to-peer support - Document caregiver interventions under the caregiver's MRN
